# Posterior instrumented correction and fusion of Scheuermann´s results in physiological reconstruction of sagittal alignment and excellent overall clinical outcome- clinical trail of 73 patients

**DOI:** 10.1186/s12891-024-08205-3

**Published:** 2025-01-28

**Authors:** M.-L. Jensch, U. Platz, M. Quante, M. Köszegvary, B. Thomsen, J. Gliemroth, C. Berlin, H. Halm

**Affiliations:** 1 Department of Spine Surgery, Ameos Clinic Eutin, Eutin, Germany; 2 Department of Neurosurgery, University Hospital Schleswig- Holstein, Lübeck, Germany; 3 Department of Spine Surgery, University Hospital Carl Gustav Carus, Dresden, Germany

**Keywords:** Scheuermann’s disease, Hyperkyphosis, SRS- 22, Equation 5D, PSF

## Abstract

**Purpose:**

The aim was to assess the clinical outcomes after posterior spinal fusion (PSF) in patients with Scheuermann’s disease (SD).

**Methods:**

SD undergoing PSF were retrospectively analyzed. Clinical outcome was determined using SRS-22- and Eq. 5D-questionaires preop and after 3, 12, 24 months after surgery. Whole spine x-rays were analyzed (preop, postop, after 6, 12, 24 months): sagittal and coronary Cobb angles, and pelvic parameters were evaluated; ideal lumbar lordosis (LL) was calculated using formula of le Huec (LL = 0.54*PI + 27.6). Surgical time, complications and blood loss were compared. Postop appearance of proximal (PJF) and distal junction failure (DJF) were calculated. Values were given as mean. Comparison with significance α = 0.05.

**Results:**

73 patients were included. SRS-22 total score and EQ5D showed significant increase from preop to two-year FU (each *p* < 0.001). Preop Thoracic kyphosis (TK) was 75.1° with significant correction to 48.5° (*p* < 0.001). LL showed mean correction from 68.2° to 46.7° (*p* < 0.001). Difference between ideal and measured LL showed improvement from − 17.2° preop to -3.3° 6 month postop, good spontaneous correction of hyperlordosis. 63% had < 10° deviation from ideal LL 6 month postoperatively, whereas only 21.4% were in this range preoperatively. No significant changes for spinopelvic parameters during FU. Complications occurred in 13,7% of cases. A low revision rate for PJF (2,7%) was necessary. Subscore mental health showed a correlation to preop TK (*p* < 0.05).

**Conclusion:**

Physiological reconstruction of sagittal alignment could be achieved in most cases (63%). Clinical FU results were convincing with significant improvement of patient’s satisfaction. Complication rate was moderate and risk of PJF after PSF low.

**Supplementary Information:**

The online version contains supplementary material available at 10.1186/s12891-024-08205-3.

## Introduction

 Scheuermann’s disease (SD) is a structural kyphotic deformity first described almost 100 years ago, which manifests in the adolescent growth phase [1]. 

The disease is defined based on radiological criteria such as wedge-shaped vertebrae of at least three consecutive vertebral bodies, irregular endplates as well as pathognomonic Schmorl nodules [1]. In SD symptoms such as back pain or neurological deficits usually do not occur before the third decade of life. In addition, restrictive pulmonary impairment is possible in patients suffering from kyphosis of more than 100 degrees. Moreover, the patient’s self-image plays a crucial role in the treatment decision [2]. 

Posterior spinal fusion (PSF) with pedicle screw dual rod systems is the most commonly performed corrective surgery for progressive kyphosis in SD that exceeds a certain degree of kyphosis [3]. 

This monocentric study aimed to investigate the effect of PSF on Health-related quality of life (HRQoL) in SD patients using the Eq. 5D and Scoliosis research score- 22 questionnaires (SRS-22) compare and quantify the effects of surgical treatment. Furthermore, it should be proved whether a physiological sagittal alignment can be restored in patients with SD-associated hyperkyphosis. Since the correction of SD typically results in spontaneous correction of compensatory lumbar hyperlordosis, it should be examined whether surgery was able to restore physiological lumbar lordosis (LL) in relation to the patient’s pelvic incidence (PI). Complications such as proximal junctional kyphosis (PJK) and distal junctional failure (DJF) were also investigated.

## Materials and methods

All data were collected within the framework of a single scoliosis center registry. The study was conducted according to the ethical principles suggested in the Declaration of Helsinki and was approved by the institutional review board of the Schoen- clinic Neustadt. All patients signed a formal consent and have agreed to the utilization of their anonymized data for scientific purposes. The database contains patient-based pre- and postoperative data as well as surgeon-based surgical data, including detailed documentation of pre- and postoperative complications. Patients having SD associated hyperkyphosis who underwent PSF and correction between 2014 and 2019 were included. In- and exclusion criteria were demonstrated in Table 1.

**Table 1:** Inclusion and exclusion criteria

Inclusion criteria:	Exclusion criteria:
- SD - Posterior spinal fusion - Periods of inclusion: 2014–2019	- Serious secondary diseases - Previous fusion operation - Additional ventral fusion/ Schwab III or higher

HRQoL was assessed using the Eq. 5D, a generic HRQoL instrument that is reported to be valid and reliable in the general population including patients who have undergone spine surgery [4]. 

Furthermore, the Scoliosis Research Society-22 (SRS-22) questionnaire was completed by the patients included in this study. The SRS-22 was developed to measure HRQoL in patients with idiopathic scoliosis aged 10 years and older [5]. Because the SRS-22 was validated in German language in 2009 and no disease-specific HRQoL instrument was available for patients suffering from SD associated hyperkyphosis, the SRS-22 was used to assess the HRQoL of those patients [6]. It can be assumed that the SRS-22 questionnaire can reliably determine function, pain, mental health, self-image and satisfaction in patients with SD.

Standing anterior-posterior (a.p.) and lateral whole spine radiographs including the pelvis (EOS^®^) in a relaxed upright position were performed preoperatively, postoperatively, and after 6, 12 and 24 months.

The diagnosis of SD was made based on the radiological criteria mentioned above. A trained observer measured radiographic parameters using a validated software (Surgimap, Nemaris Inc., New York) [7]. Modified Cobb angels were used for the determination of thoracic kyphosis (TK), which is the angle between the upper endplate of the most cranially tilted vertebra and the lower endplate of the most caudally tilted vertebra. The Stagnara angle is defined as the angle between the upper endplate of T4 and the lower endplate of T12. The lumbar lordosis (LL) was defined as the angle between the upper endplate of L1 (L4) and the top plate of S1. The sagittal vertical axis (SVA) was measured, which is defined as the distance between the C7 perpendicular and the upper rear corner of S1. Furthermore, pelvic parameters were measured as previously described, including pelvic incidence (PI), pelvic tilt (PT), and sacral slope (SS) [8]. 

**Fig. 1:**
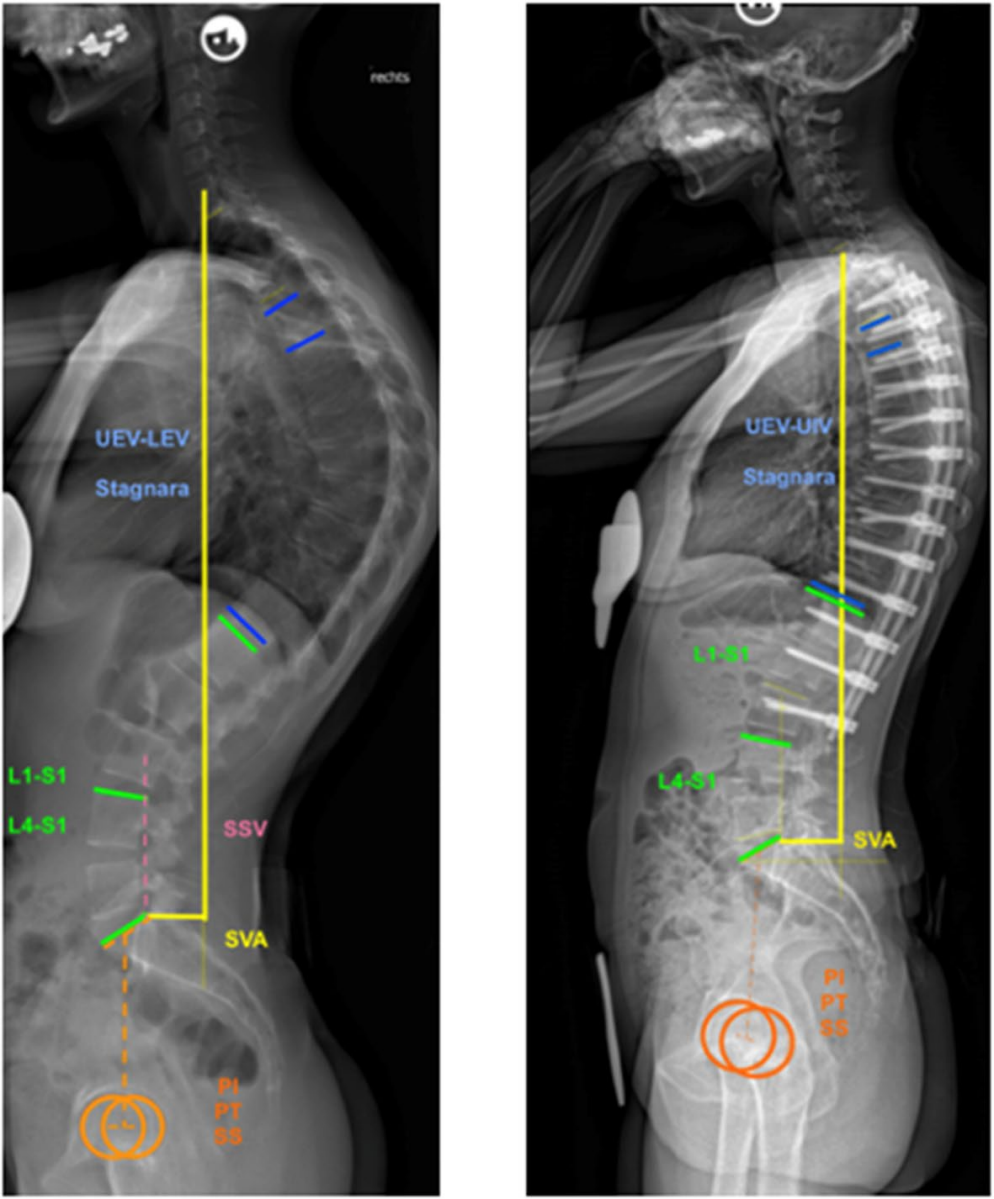
Case representation of preoperative and postoperative measurements*. *The preoperative sagittal measurement (left) and postoperative sagittal measurement (right) are depicted. The visualizations include the C7 plumb line, the kyphosis from upper end vertebra (UEV) to lower end vertebra (LEV), the Stagnara angle (T4-T12), lumbar lordosis (LL) from L1 to S1, and distal LL from L4 to S1. Additionally, measurements of pelvic tilt (PT), sacral slope (SS), and pelvic incidence (PI) are included for comprehensive evaluation

The apical vertebra of hyperkyphosis was assessed and the stable sagittal vertebra (SSV) was determined by posterior sacral vertical line (PSCV) [7, 9]. One representing case is demonstrated in Fig. 1.

All patients received a PSF using a pedicle screw-dual rod system. The correction was performed using a cantilever correction maneuver with segmental compression. To increase the flexibility before correction, each patient received multiple posterior column osteotomies (Schwab I-II) for a good correction of kyphosis.

As the proximal end of the fusion, the first vertebra above the upper end vertebra of the thoracic kyphosis was chosen (UEV/UEV + 1) to avoid proximal junctional failure (PJF). The stable sagittal vertebra (SSV) was used as the distal fusion end [10]. 

The proximal junction angle (PJA) was defined as the angle between the lower endplate of the upper instrumented vertebra (UIV) and the upper endplate two vertebrae above the UIV. Noticeable PJK was defined using the definition of Glattes et al.: The PJA should be greater than 10° and greater than 10° in comparison to the preoperative measurement according to Glattes et al. [11]

The distal junctional angle was determined synchronously caudally and used to calculate the DJF.

To analyze the physiological alignment, the ideal LL of each patient was calculated using the formula according to Le Huec (LL = 0,54*PI + 27,6) [8]. 

### Statistical analysis

Data assessment and statistical analysis were performed using Wizard Statistics &Analysis Version 1.9.38.

Quantitative variables were described using mean, standard deviation, tested for normal distribution using the Kolmogorov-Smirnov test. If there was a significant deviation from a normal distribution in the tested variables, nonparametric tests were used for further analysis. Thus, the comparison of two cohorts as independent samples was performed using the Mann-Whitney-U test and a possible relationship between two quantitative parameters was examined using Pearson’s correlation analysis. More than two cohorts were compared using the Kruskal-Wallis test.

If the quantitative variables to be tested showed no significant deviations from a normal distribution, the comparisons of two cohorts could be made using the t-test for independent samples. Post hoc analysis was performed using a Šidák correction. More than two cohorts were compared using the ANOVA.

For discrete values, comparisons were performed with a chi-square test. Statistical significance was assumed for p values less than 0.05.

## Results

73 patients could be successfully included. 25 being female and 48 males. The mean age at the time of surgery was 27.9 years and the average body-mass-index (BMI) was 24.9 kg/m^2^. The clinical follow-up (FU) rate was 84% and ranged from two to six years with a median of three years.

Already three months after surgery, an improvement of the Eq. 5D as well as the SRS-22 total score could be achieved following a continuous increase during the subsequent FU (*p* < 0.01).

**Fig. 2:**
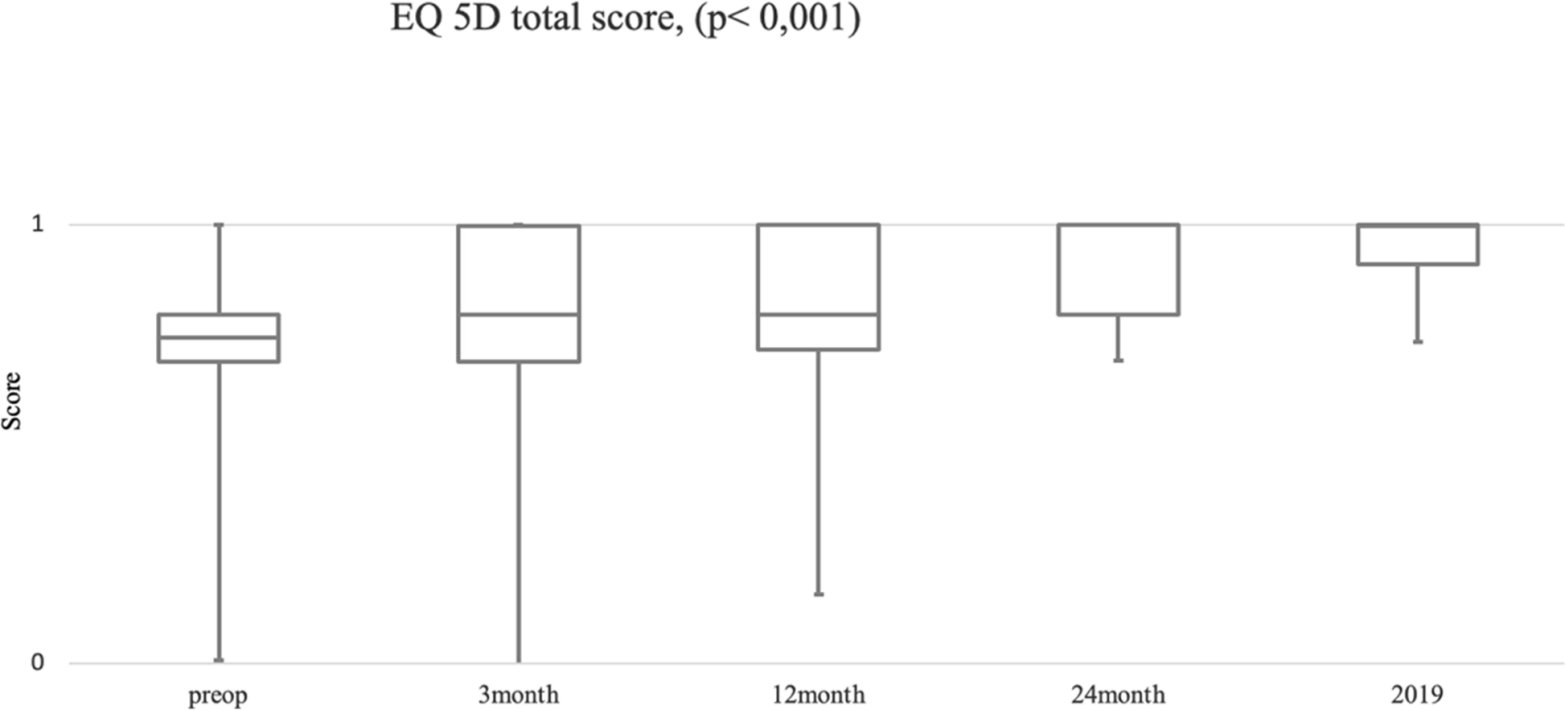
Graphical representation of the Eq. 5D -Score and FU*. *The horizontal axis shows the FU and the vertical axis shows the total score achieved Compared to preoperatively, there is a significant difference in the Eq. 5D questionnaire score postoperatively. This significant difference remains in the FU and shows a non-normal distribution (Mann-Whitney U test, *p* < 0.001).

The Eq. 5D index was 0.674 preoperatively and increased to 0.773 after three months. At the end of the clinical analysis in 2019, the Eq. 5D index was 0.957 ± 0.011 (Mann-Whitney-U-test, *p* < 0.001) (Fig. 2).

**Fig. 3:**
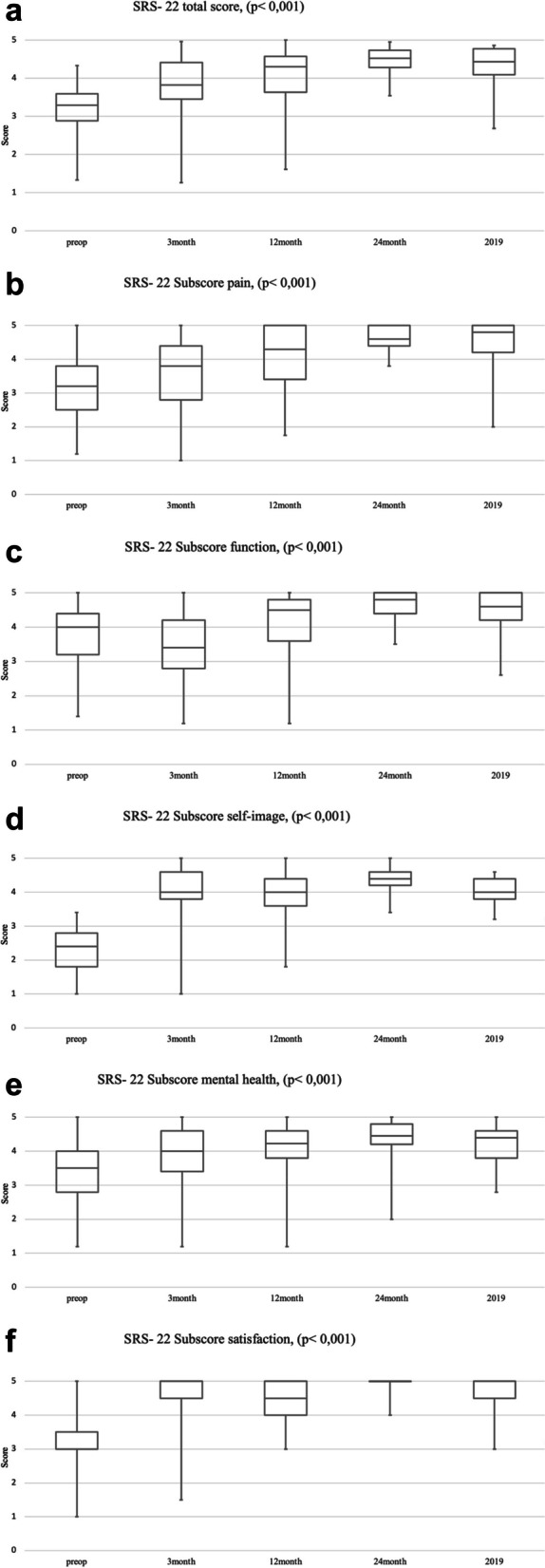
**a** Graphical representation of the SRS- 22 -Score and FU. The horizontal axis shows the FU and the vertical axis shows the total score achieved Compared to preoperatively, there is a significant difference in the SRS-22 total score postoperatively. The FU results are not normally distributed, but always remain *p* < 0.001. **b** Graphical representation of the SRS-22 Subscore pain and FU. The horizontal axis shows the FU and the vertical axis shows the total score achieved Compared to preoperatively, there is a significant difference in the SRS-22 subscore pain postoperatively. The FU results are not normally distributed, but always remain *p* < 0.001. **c **Graphical representation of the SRS-22 Subscore function and FU . The horizontal axis shows the FU and the vertical axis shows the total score achieved Compared to preoperatively, there is a significant difference in the SRS 22 subscore function postoperatively. The FU results are not normally distributed, but always remain *p*  < 0.001. **d** Graphical representation of the SRS- 22 Subscore self-image and FU. The horizontal axis shows the FU and the vertical axis shows the total score achieved compared to preoperatively, there is a significant difference in the SRS-22 subscore self-image postoperatively. The FU results are not normally distributed, but always remain *p* < 0.001. **e** Graphical representation of the SRS- 22 Subscore mental health and FU The horizontal axis shows the FU and the vertical axis shows the total score achieved. Compared to preoperatively, there is a significant difference in the SRS-22 subscore mental health postoperatively. The FU results are not normally distributed, but always remain *p *< 0.001. **f** Graphical representation of the SRS- 22 Subscore satisfaction and FU. The horizontal axis shows the FU and the vertical axis shows the total score achieved Compared to preoperatively, there is a significant difference in the SRS-22 subscore satisfaction postoperatively. The FU results are not normally distributed, but always remain p < 0.001

The overall index of the SRS-22 was 3.16 preoperatively. Already three months after surgery, there was a significant improvement to 3.76 (*p* < 0.001). At final FU, the index showed an increase to 4.32 overall. Compared to the preoperative index, there was a persistent statistically significant improvement (*p* < 0.001) (Fig. 3a).

The SRS-22 subscore pain showed a preoperative index of 3.16. After three months, there was a significant improvement to 3.5 (*p* < 0.001). The median- FU showed an improvement in pain with an index of 4.38 (Fig. 3b).

The SRS-22 subscore function and mobility showed a score decrease from 3.68 preoperatively to 3.43 in the first interval and an increase during FU (4.45, *p* < 0.001, FU 2 years) (Fig. 3c).

Preoperatively, the SRS-22 subscore of self-image showed a low value of 2.37. After three months, there was a significant improvement, which remained constantly increased over time (3.9 (*p* < 0.001)). The median- FU provided an index of 4.036 (*p* < 0.001) (Fig. 3d).

**Fig. 4:**
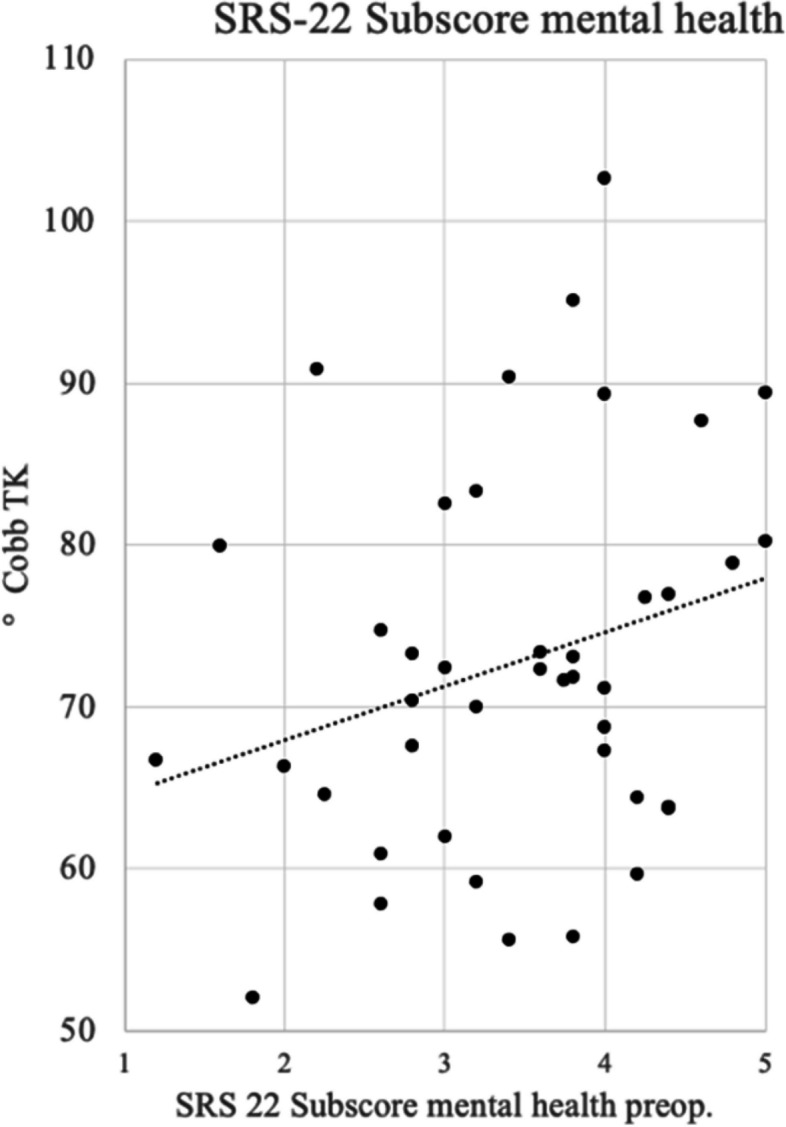
Correlation between SRS-22 subscore mental health and TK preoperatively*. *The horizontal axis shows the Score of the SRS-22 subscore mental health preoperatively; the vertical axis shows the TK (°Cobb). The dashed line shows the positive linear correlation between the two variables (*p* = 0.043)

Moreover, the SRS-22 subscore mental health showed the only positive significant correlation to the initial degree of kyphosis (*p* = 0.043) (Fig. 4). An improvement from 3.16 postoperatively to 4.06 after one year and to 4.32 at final FU was investigated (Fig. 3e).

Likewise, the SRS-22 subscore satisfaction significantly improved during FU (3.03 to 4.51 after 3 months and 4.68 at final FU (*p* < 0.001) (Fig. 3f).

Interestingly there was no correlation between the amount of correction, or the number of levels fused with patient outcome scores.

The average blood loss was 750.6 ± 49.6 ml with strong correlation to the number of fused levels (*r* = 0,273; *p* = 0,012). Surgical time was 263.7 ± 8.5 min (min), also with significant correlation to the number of fused levels (*r* = 0,304; *p* < 0.05).

The most frequently fused UIV was T2 (33 patients, 45,2%), and the most frequently lowest instrumented vertebra L3 (29 patients, 39,7%).

Using PSF TK was corrected from 75.11° preoperatively to 48.54° postoperatively (*p* < 0.001). After 6 months a loss of correction of 0,84° ±4,99° was noticed. The Stagnara angle was significantly corrected from 68.87° preoperative to 38.68° postoperative (*p* < 0,01). LL was corrected from preoperatively 68.24° to postoperatively 46.7° (*p* < 0,01).

By comparing the calculated ideal LL and the preoperatively measured LL, this study showed that a severe hyperlordosis existed preoperatively. This demonstrates that thoracic hyperkyphosis results in compensatory lumbar hyperlordosis.

The difference between the theoretically ideal LL and the measured LL was − 17.24° preoperative, and 4.8° postoperative, showing a significant correction of the severe compensatory lumbar hyperlordosis. At 6 months FU (53,42% (*n* = 39)), there was an increase of 3.32° for LL. Two-thirds of all patients (*n* = 47, 63,3%) showed less than 10° deviation from their ideal LL after six months.

Pelvic parameters showed no significant changes preoperatively, postoperatively and 6 months after surgery: PT (10,94 vs. 15,27 vs. 6month 12,89°), SS (35.24 vs. 30.76 vs. 6 months 34,68°). The measured preoperative and postoperative PT showed no significant difference from the calculated ideal PT.

On average eleven segments were fused with a positive correlation to kyphosis extent and treated levels. Consecutively, the more severe the kyphosis, the more segments had to be fused.

The overall radiological rate for the occurrence of PJK was 23.2% but only 6.8% required a surgical revision (*n* = 5). Concerning the UIV the PJK rate was 13.8% at T2, 39.1% at T3, and 20% at T4. The two revision surgeries due to PJF (PJF = PJK + neurological failure) occurred in patients with UIV at T2 (PJK = 18.9° FU 6a) and T4 (PJK = 13,4° postoperative). The DJF rate was 0% at all points of evaluation.

Overall complications occurred in 13,7% of patients (*n* = 10). Neurological complications occurred in 2,7% of the cases (*n* = 2) and wound healing disorders were observed in 2,7% (*n* = 2).

## Discussion

In summary, the study showed a significant improvement in the satisfaction of patients with SD after surgery, and this continued to increase over the long-term. Moreover, excellent and significant correction values as well as an optimization of the sagittal alignment could be achieved.

The previously stated positive postoperative correlation was also shown by Toombs et al. with an improvement of the SRS-22 in all domains from 2,8 to 4,4 points [12]. The most impressive changes were shown in the subscore self-image and mental health. Lonner et al. reviewed clinical outcome in 96 SK patients treated by PSF recorded in a multicenter database and published in 2018 [13]. Consistent with this study, patients had lower initial SRS-22 total scores, low pain, and self-image scores (3.59, 3.81 and 2.81), which improved significantly after PSF (4.31, 4.25 and 4.36) [13]. 

A recent published study by Green et al. (2020) showed a significant improvement in SRS- 22 total score in patients with surgically corrected SD to 4.2 at two-years FU (preoperative 3.6, *p* ≤ 0.000, *n* = 27). The subscore pain (preoperative 3.7; postoperative 4.4), self-image (preoperative 3.1; postoperative 4.3), and satisfaction (preoperative 3.7; postoperative 4.4) followed a similar trend. The indices of Green et al. showed comparable results to this clinical data [14]. 

Bradford et al. were the first who described results after PSF of SD in 1975. In a case study, 22 patients were included and treated surgically with a Harrington instrumentation (Harrington compression device using hooks). A mean correction of TK from 72° to 47° was achieved. In 16 patients, however, there was a loss of correction of 21° after 35 months. The more pronounced and rigid the deformity was, the higher the risk of implant-associated complications [15]. 

In the course of time, a combined anterior-posterior release and fusion was established. Later the improvement of implants and the introduction of pedicle screw-based implants led to a posterior-only approach as the preferred surgical technique for the treatment of SD [16]. Not only the complication rate but also the duration of hospital stay were lower compared with the combined fusion.

In this study, TK was corrected significantly, which corresponds with the corrections reported in the literature: Hosman et al. showed a mean correction of TK from 78.7 to 51.7° with a correction loss in the two-year FU of 1.4° [17]. Papagelopous et al. reported a correction from 68.5 to 40° (correction loss during FU 5.75°) [18]. 

In our patient population, a correction of 26.57° and a correction loss of 0,84° ±4,99°could be achieved, which corresponds with the results of the mentioned studies (Hosman et al. (27°), Papagelopous et al.(28,5°)).

Postoperatively, the radiological PJK rate in this study (23%) was superior compared to Lonner et al. with a described PJK rate of 32.1%. The isolated PJK rates of the individual fusion segments showed a significantly higher radiological PJK at the level of T3 with 39.1% (*p* = 0.023).

A low reoperation rate (*n* = 2) was demonstrated, despite the radiological PJK rate of 23%, in accordance with the equally low reoperation numbers of Lonner et al. [5]

In this study, the DJF rate was 0% at all points of the evaluation by using the SSV as the caudal fusion endpoint. Accordingly, there were no revision operations due to a DJF. In literature, there is a discussion about which end vertebra should be chosen for the LIV [19, 20]. Compared to the assumption of Cho et al., the results of this study confirm that the distal instrumented vertebra in SD should include the SSV to prevent PJF [10]. 

Another important factor is the assessment of the sagittal alignment and changes in the spinopelvic parameters. Faldini et al. were already able to show that preoperative and postoperative parameters did not show significant changes after a PSF [21]. 

In this study, a good correction was proofed without substantial loss of correction over time, corresponding well to the studies of Faldini et al. [21], Lamartina [22] and Behrbalk et al. [23], who also used a PSF described by Ponte [24, 25]. 

In particular, the PI is described as a fixed anatomical angle that defines the individual LL. By determining the PI-LL mismatch, it was able to reproduce this theory. This raises the question if a physiological alignment of the LL is possible using the calculation according to Le Huec [8]. At 6 months FU approximately two thirds deviated less than 10° from their ideal LL. However, even if the correction did not restore the calculated individual optimal sagittal balance, this did not correlate with the clinical outcome.

A direct relationship could not be shown between PI and TK in this study [26]. However, similar to Stagnara et al., it was observed that each patient has its individual sagittal spinopelvic shape, which needs to be taken into account during surgery to achieve a spinopelvic balance. This relationship is reflected in the individual but anatomically constant parameter of PI [9]. 

### Limitations

A limitation of this study is the low radiological FU rate after 6 months (53,42%, *n* = 39) and 12 months (23,4%, *n* = 17). Comparable studies (Hosman et al. Group A *n* = 16, Group B *n* = 17 [17], Papagelopous et al. *n* = 21 [18]) showed a much smaller overall study size, but a higher FU after 1 year (FU 1a = 100%).

## Conclusion

Through clinical analysis of the SRS-22 and Eq. 5D questionnaires a significant positive effect on patients’ previously impaired mental health after surgery was demonstrated. Good long-term clinical outcomes were achieved in all investigated domains and a high postoperative patient satisfaction with a SRS-22 subscore of satisfaction 4.68 at two years FU was evident.

A physiological reconstruction of the sagittal profile after PSF of SD is possible. Comparable to other studies, it was shown that the preoperative compensation mechanism takes place by lumbar hyperlordosis. Two-thirds of all patients show less than 10° deviation from their ideal LL after six months postoperatively.

A low complication rate (13.7%, *n* = 10) with a rare occurrence of PJF (2,7%) and DJF (0%) requiring revision could be achieved.

## Supplementary Information

Additional file 1.

## Data Availability

The raw data is provided in a supplementary file.

## References

[1] Sørensen KH. Scheuermann’s juvenile kyphosis: clinical appearances, radiography, aetiology, and prognosis. Munksgaard; 1964.

[2] Lowe TG. Scheuermann disease. J Bone Joint Surg Am. 1990;72(6):940–5. 2195036

[3] Mehdian H, Khurana A, Stokes OM. Posterior spinal fusion and correction of Scheuermann kyphosis. Eur Spine J. 2015;24(S5):660–3.

[4] Group E. EuroQol–a new facility for the measurement of health-related quality of life. Health Policy. 1990;16(3):199–208. 10109801 10.1016/0168-8510(90)90421-9

[5] Asher M, Min Lai S, Burton D, Manna B. Discrimination validity of the scoliosis research society-22 patient questionnaire: relationship to idiopathic scoliosis curve pattern and curve size. Spine (Phila Pa 1976). 2003;28(1):74–8. 12544960 10.1097/00007632-200301010-00017

[6] Niemeyer T, Schubert C, Halm HF, Herberts T, Leichtle C, Gesicki M. Validity and reliability of an adapted German version of scoliosis research society-22 questionnaire. Spine (Phila Pa 1976). 2009;34(8):818–21. 19365251 10.1097/BRS.0b013e31819b33be

[7] Lafage R, Ferrero E, Henry J, Challier V, Diebo B, Liabaud B et al. Validation of a new computer-assisted tool to measure spino-pelvic parameters. Spine J. 2015;15(12):2493–502. 10.1016/j.spinee.2015.08.067 26343243

[8] Le Huec JC, Aunoble S, Philippe L, Nicolas P. Pelvic parameters: origin and significance. Eur Spine J. 2011;20(Suppl 5):564–71. 21830079 10.1007/s00586-011-1940-1 PMC3175921

[9] Stagnara P, De Mauroy JC, Dran G, Gonon GP, Costanzo G, Dimnet J, et al. Reciprocal angulation of vertebral bodies in a sagittal plane: approach to references for the evaluation of kyphosis and lordosis. Spine (Phila Pa 1976). 1982;7(4):335–42. 7135066 10.1097/00007632-198207000-00003

[10] Cho KJ, Lenke LG, Bridwell KH, Kamiya M, Sides B. Selection of the optimal distal fusion level in posterior instrumentation and fusion for thoracic hyperkyphosis: the sagittal stable vertebra concept. Spine (Phila Pa 1976). 2009;34(8):765–00. 19365243 10.1097/BRS.0b013e31819e28ed

[11] Glattes RC, Bridwell KH, Lenke LG, Kim YJ, Rinella A, Edwards C 2. Proximal junctional kyphosis in adult spinal deformity following long instrumented posterior spinal fusion: incidence, outcomes, and risk factor analysis. Spine (Phila Pa 1976). 2005;30(14):1643–9. 16025035 10.1097/01.brs.0000169451.76359.49

[12] Toombs C, Lonner B, Shah S, Samdani A, Cahill P, Shufflebarger H, et al. Quality of Life Improvement following surgery in adolescent spinal deformity patients: a comparison between Scheuermann Kyphosis and adolescent idiopathic scoliosis. Spine Deform. 2018;6(6):676–83. 30348343 10.1016/j.jspd.2018.04.009

[13] Lonner BS, Parent S, Shah SA, Sponseller P, Yaszay B, Samdani AF, et al. Reciprocal changes in Sagittal Alignment with Operative Treatment of Adolescent Scheuermann kyphosis-prospective evaluation of 96 patients. Spine Deform. 2018;6(2):177–84. 29413741 10.1016/j.jspd.2017.07.001

[14] Green C, Brown K, Caine H, Dieckmann RJ, Rathjen KE. Prospective comparison of patient-selected operative Versus Nonoperative Treatment of Scheuermann Kyphosis. J Pediatr Orthop. 2020;40(8):e716–9. 32341242 10.1097/BPO.0000000000001576

[15] Bradford DS, Moe JH, Montalvo FJ, Winter RB. Scheuermann’s kyphosis. Results of surgical treatment by posterior spine arthrodesis in twenty-two patients. J Bone Joint Surg Am. 1975;57(4):439–48. 1141252

[16] Horn SR, Poorman GW, Tishelman JC, Bortz CA, Segreto FA, Moon JY, et al. Trends in Treatment of Scheuermann Kyphosis: a study of 1,070 cases from 2003 to 2012. Spine Deform. 2019;7(1):100–6. 30587300 10.1016/j.jspd.2018.06.004 PMC7102192

[17] Hosman AJ, Langeloo DD, de Kleuver M, Anderson PG, Veth RP, Slot GH. Analysis of the sagittal plane after surgical management for Scheuermann’s disease: a view on overcorrection and the use of an anterior release. Spine (Phila Pa 1976). 2002;27(2):167–75. 11805663 10.1097/00007632-200201150-00009

[18] Papagelopoulos PJ, Klassen RA, Peterson HA, Dekutoski MB. Surgical treatment of Scheuermann’s disease with segmental compression instrumentation. Clin Orthop Relat Research^®^. 2001;386:139–49. 10.1097/00003086-200105000-00018 11347827

[19] Gong Y, Yuan L, He M, Yu M, Zeng Y, Liu X, et al. Comparison between stable Sagittal Vertebra and First Lordotic Vertebra Instrumentation for Prevention of Distal Junctional Kyphosis in Scheuermann Disease: systematic review and Meta-analysis. Clin Spine Surg. 2019;32(8):330–6. 30762837 10.1097/BSD.0000000000000792

[20] Yanik HS, Ketenci IE, Coskun T, Ulusoy A, Erdem S. Selection of distal fusion level in posterior instrumentation and fusion of Scheuermann kyphosis: is fusion to sagittal stable vertebra necessary? Eur Spine J. 2016;25(2):583–9. 26195078 10.1007/s00586-015-4123-7

[21] Faldini C, Traina F, Perna F, Borghi R, Martikos K, Greggi T. Does surgery for Scheuermann kyphosis influence sagittal spinopelvic parameters? Eur Spine J. 2015;24(Suppl 7):893–7. 26441254 10.1007/s00586-015-4253-y

[22] Lamartina C. Posterior surgery in Scheuermann’s kyphosis. Eur Spine J. 2010;19(3):515–6. 20238438 10.1007/s00586-010-1351-8 PMC7571621

[23] Behrbalk E, Uri O, Parks RM, Grevitt MP, Rickert M, Boszczyk BM. Posterior-only correction of Scheuermann kyphosis using pedicle screws: economical optimization through screw density reduction. Eur Spine J. 2014;23(10):2203–10. 25103951 10.1007/s00586-014-3472-y

[24] Geck MJMA, Ponte A, Shufflebarger HL. The Ponte procedure: posterior only treatment of Scheuermann’s kyphosis using segmental posterior shortening and pedicle screw instrumentation. J Spinal Disord Tech. 2007;20(8):586–93. 10.1097/BSD.0b013e31803d3b16 18046172

[25] Haher TR, et al. Section II The Thoracic Spine, Posterior Column Shortening for Scheuermann’s Kyphosis: An Innovative One-Stage Technique, Surgical Techniques for the Spine. 2003;(1):107-9.

[26] Lonner BS, Newton P, Betz R, Scharf C, O’Brien M, Sponseller P, et al. Operative management of Scheuermann’s kyphosis in 78 patients: radiographic outcomes, complications, and technique. Spine. 2007;32(24):2644–52. 18007239 10.1097/BRS.0b013e31815a5238

