# Supplementary material for: Posterior instrumented correction and fusion of Scheuermann´s results in physiological reconstruction of sagittal alignment and excellent overall clinical outcome- clinical trail of 73 patients
Source: BMC Musculoskelet Disord. 2025 Jan 28;26:90. doi: 10.1186/s12891-024-08205-3 (PMC11773709; doi:10.1186/s12891-024-08205-3)
Supplement: Supplementary file 1 — Additional file 1. [file 12891_2024_8205_MOESM1_ESM.docx]

Raw data

Clinical outcome:

|  | preoperativ | 3month | 12month | 24month | 2019 |
| --- | --- | --- | --- | --- | --- |
| EQ- 5D |  | | | | |
| Median | 0,7425 | 0,796 | 0,796 | 1 | 0,999 |
| 1. Quartile | 0,689 | 0,689 | 0,716 | 0,796 | 0,91 |
| 3. Quartile | 0,796 | 0,999 | 1 | 1 | 1 |
| SRS- 22- score |  |  |  |  |  |
| Median | 3,286365 | 3,81818 | 4,295455 | 4,522725 | 4,432 |
| 1. Quartile | 2,88889 | 3,45455 | 3,63636 | 4,28571 | 4,090909091 |
| 3. Quartile | 3,59091 | 4,40909 | 4,57143 | 4,72727 | 4,772727273 |
| SRS- 22 pain |  |  |  |  |  |
| Median | 3,2 | 3,8 | 4,3 | 4,6 | 4,8 |
| 1. Quartile | 2,5 | 2,8 | 3,4 | 4,4 | 4,2 |
| 3. Quartile | 3,8 | 4,4 | 5 | 5 | 5 |
| SRS- 22 function |  |  |  |  |  |
| Median | 4 | 3,4 | 4,5 | 4,8 | 4,6 |
| 1. Quartile | 3,2 | 2,8 | 3,6 | 4,4 | 4,2 |
| 3. Quartile | 4,4 | 4,2 | 4,8 | 5 | 5 |
| SRS- 22 psych |  |  |  |  |  |
| Median | 3,5 | 4 | 4,225 | 4,45 | 4,4 |
| 1. Quartile | 2,8 | 3,4 | 3,8 | 4,2 | 3,8 |
| 3. Quartile | 4 | 4,6 | 4,6 | 4,8 | 4,6 |
| SRS- 22 self image |  |  |  |  |  |
| Median | 2,4 | 4 | 4 | 4,4 | 4 |
| 1. Quartile | 1,8 | 3,8 | 3,6 | 4,2 | 3,8 |
| 3. Quartile | 2,8 | 4,6 | 4,4 | 4,6 | 4,4 |
| SRS- 22 satisfaction |  |  |  |  |  |
| Median | 3 | 5 | 4,5 | 5 | 5 |
| 1. Quartile | 3 | 4,5 | 4 | 4,99 | 4,5 |
| 3. Quartile | 3,5 | 5 | 5 | 5 | 5 |

Radiological raw data

|  | preoperativ | postoperativ | 6month | 12month | 24month |
| --- | --- | --- | --- | --- | --- |
| Variable | **mean ± standard deviation** | | | | |
| TK (°) | 72,37 ± 10,99 | 43,04 ± 9,65 | 41,35 ± 9,85 | 39,25 ± 7,46 | 27,48±8,92 |
| Stagnara (°) | 68,87 ± 11,34 | 38,68 ± 9,63 | 36,88 ± 9,93 | 34,62 ± 7,99 | 26,35±9,08 |
| UEV-LEV (°) | 75,71 ± 11,23 | 48,54 ± 10,72 | 55,51 ± 14,57 | 58 ± 12,24 | 56,35±10,94 |
| LL L1 – S1 (°) | -68,42 ± 13,49 | -46,69 ± 12,74 | -55,69±13,78 | -56,06±11,59 | -49,3±8,86 |
| LL L4 – S1 (°) | -41,06 ± 9,11 | -32,04 ± 10,52 | -38,90± 8,27 | -38,64±9,89 | -41,8±6,1 |
| SVA (mm) | 13,57 ± 34,21 | 23,04 ± 26,19 | -5,39 ± 36,61 | -15,32 ± 32,52 | 5,53±30,13 |
| PT (°) | 10,93 ± 10,78 | 15,27 ± 10,67 | 13,04 ± 12,01 | 11,84 ± 9,72 | 8,67±4,38 |
| PI (°) | 46,17 ± 15,32 | 46,05 ± 15,18 | 48,3 ± 14,93 | 42,78±15,95 | 46,87±9,93 |
| SS (°) | 35,24 ± 12,11 | 30,76 ± 10,98 | 35,29 ± 11,31 | 30,91 ± 9,84 | 38,23±5,65 |
| Coronal deviation (mm) | 4,1 ± 11,84 | -5,8 ± 12,09 | 4,09 ± 9,96 | -1,72 ± 13,42 | -7,5±30,13 |
| Pelvic level(mm) | 5,75 ± 5,87 | 5,84 ± 5,89 | 5,44 ± 6,47 | 4,21 ± 5,16 | 2,25 ± 4,5 |
| Shoulder level (mm) | 8,49 ± 6,99 | 8,54 ± 7,27 | 5,84 ± 5,41 | 5,72 ± 3,76 | 9,23 ± 6,05 |
| UIV+2 (°) | - | 9,05 ± 6,68 | 14 ± 9,66 | 16,82± 10,90 | 17,68±11,08 |
| LIV+2 (°) | - | -17,49 ± 10,21 | -21 ± 11,02 | -24,83±9,82 | -4,05 ± 12,76 |
